# Impact of the Proactive Rounding Team on Rapid Response System During COVID-19 Pandemic: A Retrospective Study From an Italian Medical Center

**DOI:** 10.7759/cureus.24432

**Published:** 2022-04-24

**Authors:** Agostino Roasio, Eleonora Costanzo, Giorgio Bergesio, Stefano Bosso, Sandro Longu, Franca Zapparoli, Stefano Bertocchini, Germana Forno, Alessandro Fogliati, Maria Teresa Novelli

**Affiliations:** 1 Anesthesia and Critical Care, Cardinal Massaia Hospital, Asti, ITA; 2 Sciences of Public Health and Pediatrics, University of Turin, Asti, ITA; 3 Pulmonology and Critical Care, Cardinal Massaia Hospital, Asti, ITA; 4 Intensive Care Unit, Cardinal Massaia Hospital, Asti, ITA

**Keywords:** sars-cov-2, in-hospital cardiac arrest, covid-19, medical emergency team, rapid response team

## Abstract

Objective: During the coronavirus disease 2019 (COVID-19) pandemic a proactive rounding (PR) team was introduced in our clinical practice in order to recognize the clinical deterioration of the patient as soon as possible. This study aimed to evaluate the impact of the PR team on the rapid response system (RRS) workload with particular regard to the activity carried out, the mode of intervention, and the outcome of patients.

Methods: In this retrospective study, the first period before the activation of the PR team (March 1, 2019, to February 29, 2020) and the second period after its activation (March 1, 2020, to March 1, 2021) were compared.

Results: A total of 406 inpatient RRS activations were collected. The medical emergency team (MET) dose was 13 and 12.2 activations/1000 admitted patients per year while the incidence of unexpected cardiac arrests was 3.8 and 2.6 events/1000 admitted patients per year (p=0.10). MET response time was longer in the second period (3.5±1.6 minutes vs 4.5±2.6 minutes p<0.01). We recorded more RRS activations for medical patients than surgical ones; MET was activated more frequently by physicians than nurses and for less severe criteria. Patients admitted to the intensive care unit had lower Simplified Acute Physiology Score II (SAPS II) scores.

Conclusions: The PR team introduced during the COVID-19 pandemic did not increase the RRS workload. In addition, it allowed an earlier activation of the MET, especially by physicians.

## Introduction

The deterioration of the vital parameters of the patient is often an underestimated event that, in the most severe cases, precedes in-hospital cardiac arrest (IHCA) [1]. During the last two decades an organized system, called rapid response system (RRS), has been set up to recognize as early as possible the worsening of the patient and prevent the most catastrophic event [2-4]. It consists of four components, similar to arms, that interact with each other through a network of relationships [5]. The afferent arm is the staff monitoring the patient, tasked with alerting the system in case of clinical deterioration. The efferent arm is the team that reacts to the emergency also known as medical emergency team (MET) or rapid response team (RRT). In Italy, it is usually composed of an anesthesiologist and a nurse who works in the intensive care unit (ICU) [6,7]. In addition, there are the system administration service and the quality management service with the purpose of maintaining and constantly improving the quality of the entire system [8]. When promptly activated, a consolidated RRS constitutes a safety standard in the management of hospitalized patients, allowing to reduce cardiac arrests and unexpected ICU admissions [9,10]. Conversely, a delay in the activation of RRS has a negative impact on the patient's clinical course [11,12]. To obtain an increasingly early warning of the RRS, some studies proposed management called proactive rounding, which is able to intercept as quickly as possible a clinical deterioration with a positive effect on the outcome [13,14].

At the beginning of 2020, the severe acute respiratory syndrome coronavirus-2 (SARS-CoV-2) infection caused severe problems to healthcare facilities all around the world, heavily impacting the organization and workload of emergency services and subsequently ICUs [15-18]. Additionally, an increase in intra-hospital emergencies and cardiac arrests with worsened outcomes was observed [19]. To our knowledge, there are no data in the literature about the impact of a proactive rounding team (PR team) on RRS.

This study aimed to evaluate the impact of the PR team introduced in our clinical practice during the COVID-19 pandemic on RRT with particular regard to the activity carried out, the mode of intervention, and the outcome for patients.

## Materials and methods

We performed a single-center retrospective before-after study. We compared RRT activations before and after the introduction of the PR team during the COVID-19 pandemic. The period of time considered for the analysis spans from March 1, 2019, to March 1, 2021. This timeframe has been divided into two phases: the first phase, spanning from March 1, 2019, to February 29, 2020, covers the period before the COVID-19 pandemic in Italy and the introduction of PR team (before PR team phase), while the second phase (from March 1, 2020, to March 1, 2021) regards RRS calls after the introduction of PR team (after PR team phase).

Setting

A second level 500-bed Italian hospital with 10 ICU beds increased to 26 during the pandemic. The RRT is available seven days a week, 24 hours a day, intervening both on calls from hospitalized and non-hospitalized people (staff, visitors, and outpatients). It can be activated by doctors, nurses, or non-healthcare personnel by calling the unique emergency number (2222). In our setting the MET is composed of an anesthesiologist and critical care nurse. There are several RRT activation systems based on single parameter, multiple parameters, or aggregated scores such as the National Early Warning Score [20,21]. Our hospital adopted the multiple parameters alert criteria with color codes. It is a simple and easy coding system that gives each parameter a different weight according to their severity by dividing them into red or yellow codes. The red code indicates an imminent life-threatening clinical situation while the yellow code is related to a serious risk of deterioration (Table 1).

**Table 1:** Criteria differentiation by color code. Bpm: beat per minute

Alert criteria	Clinical parameter
Red code	Obstructed or at risk airways
	Absent consciousness
	Respiratory arrest
	Cardiac arrest
Yellow code	Respiratory distress
	Respiratory rate > 30 breaths/min or < 8 breaths/min
	Sudden oxygen saturation < 90%
	Heart rate < 40 bpm or > 130 bpm
	Systolic blood pressure < 90 mmHg
	Sudden alteration of consciousness
	Sudden cyanosis if oximetry not available
	Blood loss ≥ 200 ml/h
	Body temperature > 38.5°C or < 35°C

Two yellow codes or one red code are required to activate RRS. The measure of RRT utilization rate is obtained with the MET dose (the ratio between the number of inpatients RRT activations every 1000 admissions per year). Another important data related to in-hospital emergencies is the incidence of IHCA referred to as the ratio between the number of unexpected in-hospital cardiac arrests every 1000 admitted patients per year [22,23].

The PR team was introduced in our hospital during the coronavirus disease 2019 (COVID-19) pandemic. The first SARS-CoV-2 infected patient was admitted to the hospital on March 1, 2020, and therefore we consider this date as the beginning of the pandemic period in our hospital. Since that date, an ad hoc PR team, consisting of a critical care physician and a respiratory disease nurse, has been established. The aim of the team was to set up scheduled checks of the inpatients, especially those suffering from SARS-CoV-2 pneumonia. The team evaluated the patient’s clinical parameters twice a day, in a timeframe ranging from 8 a.m. to 8 p.m.

Inclusion and exclusion criteria

We collected all the inpatient RRT activations included in the Italian Cardiac Arrest Registry endorsed by the Italian Resuscitation Council, recording both IHCA and other emergencies. We excluded outpatient RRT calls or largely incomplete data.

Primary and secondary end-points

The main endpoint, primary, of the study is to evaluate how the PR team impacted the RRT’s workload comparing the MET dose and the incidence of IHCA in the period before and after its introduction. The secondary end-points are the research assesses the RRT response times, type of admitted patients, staff activating the RRT, and call criteria. Finally, we evaluated the RRT outcome considering the number of ICU admission, their severity, and hospital mortality.

Data collected are inpatient RRT calls, type of emergency (IHCA or other emergencies), the number of admitted patients which allowed to derive the MET dose, and the incidence of IHCA; demographic data such as age (years), gender (male or female), type of hospitalization (medical or surgical), if SARS-CoV-2 affected patients; RRT response time interval (minutes elapsed from the call to the arrival of the MET); data referring to the RRT call such as personnel who activated the service (physician, nurse, or non-healthcare operator) and the call criterion (red code or yellow code); RRT outcome data such as ICU admission, the severity of ICU admitted patient using the Simplified Acute Physiology Score II (SAPS II) and the Sequential Organ Failure Assessment (SOFA); hospital mortality.

Ethics and statistics

The study was carried out as a retrospective collection using anonymously stored data, hence the consensus of the ethics committee was not considered necessary. The data have been processed in compliance with the current national laws on patient privacy. The numerical data are presented as mean±standard deviation (SD) while ordinal data as percentage. The comparison between the two groups (before PR team and after PR team) was obtained using the Student's t-test for numerical data, while ordinal values were compared with the χ2 test. We used Excel 2010 with the XLSTAT® expansion (New York City, NY: Addinsoft). A p-value less than 0.05 was considered statistically significant.

## Results

A total of 406 inpatient RRT calls were included in our study; 207 RRT calls were realized during the first phase, while 199 after the introduction of PR team. The distribution of cardiac arrest and other emergencies did not demonstrate significant changes in the two periods of the study (cardiac arrest 13.6% vs 11.5% and other emergencies 86.4% vs 88.5%, p=0.13).

The RRT workload carried out before and after activation of the PR team did not significantly change. In fact, the number of annual hospitalizations (15,867 admitted patients before PR team introduction and 16,283 after its introduction) allowed to obtain a MET dose of 13 calls every 1000 admitted patients during the first phase and 12.2 calls every 1000 admitted patients during the second phase. The incidence of IHCA was 3.8 cases during the first phase and 2.6 cases in the after PR team period (p=0.1). Patients for whom RRS was activated were similar for age (72.7±15.8 vs 70.5±13.6 years, p=0.13) and gender (males 66.7% vs 67.8% and females 33.3% vs 32.2%, p=0.80). Concerning the type of admission, we recorded significantly more medical patients (82.6% vs 90.9%) than surgical ones (17.4% vs 9.1%) (p=0.02). There were 109/199 (50.7%) COVID-19 patients.

The RRT response time interval increased significantly in the second phase of the study (3.5±1.6 minutes vs 4.5±2.6 minutes p<0.01). The RRT was activated more frequently by medical staff in the second phase of the study (28% vs 47.3%), than by nursing staff who, in turn, activated the MET more often in the first phase (63.3% vs 47.3%) (p<0.01). Calls from non-healthcare professionals did not vary significantly (8.7% vs 5.4%, p=0.21). With regard to the RRT alert criteria, there was a lower incidence of red codes (42.1% vs 30.7%) and a higher incidence of yellow codes (57.9% vs 69.3%) (p=0.02). The outcome data, summarized in Table 2, show no significant change in ICU admissions and in-hospital mortality. It is to be noted that patients admitted to ICU after PR team introduction had significantly lower SAPS II figures.

**Table 2:** Data concerning ICU admission and outcome. ICU: intensive care unit, SAPS II: Simplified Acute Physiology Score II, SOFA: Sequential Organ Failure Assessment

	Before PR team	After PR team	p-Value
ICU admissions	14%	19.6%	0.13
SAPS II (mean±SD)	45±19	37±9	0.03
SOFA (mean±SD)	6±4	8±3	0.24
Hospital mortality	59.4%	57.3%	0.33

## Discussion

The RRT was set up with the aim of intervening on the patient's deterioration as early as possible, allowing a quick diagnosis and treatment [3]. It is currently considered as the highest standard in medical care and healthcare safety [2]. ICU staff is the cornerstone of the management of the MET, being the efferent arm and the most operational of the entire system. The recent pandemic due to SARS-CoV-2 virus has subjected the ICU personnel to arduous work and new challenges [24,25]. Within this context, a team with a proactive function was introduced to recognize as early as possible the worsening of patients and to optimize the use of resources available. Additionally, the literature suggests that the introduction of a proactive rounding team may be able to determine a reduction in cardiac arrests and unexpected ICU admissions [26]. These positive effects are nonetheless associated with a more frequent activation of the MET and a subsequently heavier workload [27]. Our study did not show an increase in the RRS workload following the activation of the PR team. On the contrary, the MET workload as indicated by MET dose remained substantially stable passing from 13 to 12.2 every 1000 admitted patients per year. In fact, the implementation of the PR team might have led to more accurate MET calls, by preceding it with a deep clinical evaluation. Our study did not demonstrate any significant variation of unexpected events between the first and second periods (3.8 and 2.6 cases every 1000 admitted patients per year, respectively). Our results are countertrend compared with other studies released during COVID-19 pandemic where an increase in IHCA rate was reported [21]. This may be related to a positive effect of the proactive evaluation realized in the second period of our analysis. This study showed that the RRS was activated for patients similar in demographic characteristics (age and gender), but different for the type of in-hospital admissions. In the second phase, during COVID-19 pandemic, MET treated mostly medical admitted patients rather than surgical ones. This result may be due to the drastic reduction in surgical activity amidst the emergency sparked from the spread of the virus. In addition, we demonstrated a significant increase in RRS response time during the second period of the study (3.5±1.6 vs 4.5±2.6 minutes, p<0.01). This longer response time may be explained by the need to wear Personal Protective Equipment and more burdensome isolation where ICU staff worked. In our study, the RRS calls differed statistically both for the operator who activated the system, as well as for the alert criteria. A greater number of activations by medical staff was indeed recorded (28% vs 47.3%), for RRT calls by the nursing staff (63.3% vs 47.3%) (p<0.01). To cope with increased hospitalizations during the pandemic, more physicians were required in wards prepared to care for such affected patients. In order to deal with personnel shortage, young and less skilled personnel, or physician coming from other specialties, were required to treat and manage COVID-19 patients. This led to very unusual and difficult working conditions for doctors who were forced to face pathologies beyond their skills (in particular serious respiratory diseases) and often had to work in an unconventional environment for setting and staff. In these cases, the PR team helped clinicians activate the RRS. The research points out also a significant variation in the alert criteria, presenting a reduction in red alert codes (42.1% vs 30.7%), whereas there was an increase in yellow codes (57.9% vs 69.3%) (p=0.02). This result could be indicative of an earlier RRT activation when the patients’ conditions are still less severe. The introduction of the PR-Team during the pandemic has also been aimed at optimizing patients’ access to the ICU in order to balance resources and requests [18]. In fact, our research showed a non-significant increase in ICU admission from 14% to 19.6% (p=0.13). Also, the absolute value of ICU admitted patients (19.6%) is lower than data reported (30%) [28,29]. With regard to the severity of ICU admitted patients we demonstrated a significant reduction in SAPS II in the second phase of the study (45±19 vs 39±9, p=0.03). This result may confirm a less severe clinical picture at ICU admission for patients treated after the introduction of the PR-Team.

Finally, intra-hospital mortality did not vary significantly over the two periods considered. Our result is in line with other experience [30]. In fact, in the cited study, assessing data from over 130 European hospitals, the hospital mortality for COVID-19 patients affected by severe ARDS is recorded at 53%, similar to our work. Some limitations affected our study such as the type of analysis (single-center) and the different work conditions. In fact, the introduction of the PR team took place during the pandemic when there was a different work setting.

## Conclusions

The introduction of the PR team into clinical practice permitted an earlier recognition of clinical deterioration and optimized ICU resources without a significant change in the activation and workload of RRS. It was able to counteract the increases of IHCA expected during the pandemic. In addition, the PR team supported clinicians in general departments in the phase of activation of the RRS (especially the medical staff), anticipating the moment of alert in a phase of less severe clinical situations.
